# Quality of Life after Surgical Treatment of Brain Tumors

**DOI:** 10.3390/jcm11133733

**Published:** 2022-06-28

**Authors:** Agnieszka Królikowska, Karolina Filipska-Blejder, Renata Jabłońska, Beata Haor, Anna Antczak-Komoterska, Monika Biercewicz, Lech Grzelak, Marek Harat, Robert Ślusarz

**Affiliations:** 1 Neurological and Neurosurgical Nursing Department, Faculty of Health Science, Collegium Medicum in Bydgoszcz, Nicolaus Copernicus University in Toruń, Łukasiewicza 1 Street, 85-821 Bydgoszcz, Poland; karolinafilipskakf@gmail.com (K.F.-B.); renjab_1@wp.pl (R.J.); beata.haor@interia.pl (B.H.); robert_slu_cmumk@wp.pl (R.Ś.); 2 Institute of Health Science, The State Vocational University in Włocławek, Obrońców Wisły 1920r. 21/23 Street, 87-800 Włocławek, Poland; anna.antczak-komoterska@puz.wloclawek.pl; 3 Clinic of Geriatrics, Faculty of Health Science, Collegium Medicum in Bydgoszcz, Nicolaus Copernicus University in Toruń, Skłodowskiej 9 Street, 85-094 Bydgoszcz, Poland; monika_bierc_cmumk@wp.pl; 4 Department of Neurosurgery of the Specialist Municipal Hospital of Nicolaus Copernicus in Toruń, Batorego 17-19 Street, 87-100 Toruń, Poland; lechg7@gmail.com; 5 Department of Neurosurgery, The 10th Military Research Hospital, Powstańców Warszawy 5 Street, 85-681 Bydgoszcz, Poland; marek.harat@cm.umk.pl

**Keywords:** quality of life, brain tumor, surgery

## Abstract

Quality of life is one of the parameters that characterize the success of brain tumor treatments, along with overall survival and a disease-free life. Thus, the main aim of this research was to evaluate the quality of life after the surgical treatment of brain tumors. The research material included 236 patients who were to undergo surgery for brain tumors. The participants completed the quality of life questionnaires EORTC QLQ-C30 (version 3.0) and EORTC QLQ-BN20 on the day of admission to the department, on the fifth day after the removal of the brain tumor, and thirty days after the surgical procedure. Descriptive statistics, Student’s *t*-test, the Kruskal–Wallis test, the Shapiro–Wolf test, ANOVA, and Fisher’s least significant difference post hoc test were performed. The mean score of the questionnaire before the surgical procedure amounted to 0.706, 5 days after surgery it amounted to 0.614, and 30 days after surgery to 0.707. The greatest reduction in the quality of life immediately after the procedure was observed in patients with low-grade glial tumors (WHO I, II) and extracerebral tumors (meningiomas and neuromas). Thirty days after surgery, an improvement in the quality of life was observed in all included groups. The greatest improvement was recorded in the group of patients operated on for meningioma and neuroblastoma, and the lowest in patients treated for metastatic tumors. Contemporary surgical procedures used in neurosurgery reduce the quality of life in patients with brain tumors only in the early postoperative period. Histopathological diagnoses of these tumors impact the quality of life of patients.

## 1. Introduction

Intracranial neoplasms include neuroepithelial changes, cranial and spinal nerves, meninges, lymphomas, primary germ cells, Turkish saddle, and metastatic neoplasms [1]. According to data presented by the Central Brain Tumor Registry of the United States, malignant tumors account for 29.7% of all brain and central nervous system (CNS) tumors. The most common malignant tumors are glioblastoma (48.6%) and diffuse/anaplastic astrocytoma (11.6%). Among nonmalignant tumors, the most common are meningioma (53.9%), pituitary tumors (24%), and nerve sheath tumors (12.1%) [2]. In Poland, the most commonly diagnosed tumors include meningioma (25%), pituitary tumors (25%), and glioblastoma (15%) [3]. Low-grade glioma occurs most often in young and middle-aged people, while malignant glial tumors, especially glioblastoma, occur in older adults. The median survival for patients with low-grade tumors may be more than 10 years, and for patients with high-grade tumors, it ranges from 1 to 3 years. For glioblastoma (the most common primary brain tumor in adults), the median progression-free survival is 9 months and the overall survival is 19 months. The radicality of tumor removal is of great importance in surgical treatment. However, sometimes tumors are located in parts of the brain that prevent complete resection, as this approach carries a high risk of damaging healthy brain tissue. In these situations, as much of the tumor (as is safe) is removed. Even partially removing the tumor can help reduce symptoms and reduce the pressure on nearby parts of the brain. These neoplasms, despite surgical treatment, radiotherapy, and chemotherapy, cause recurrences, and cancer progression leads to death in most cases. Unfortunately, glioma is still an incurable disease. Hence, in this group of patients, the aim of treatment is to extend life (for as long as possible) and maintain a good quality of life [1,2,3,4,5]. There is also a large group of meningiomas, which are usually benign lesions (88–95%), more common in women (2:1), originating from meningothelial tissues [6]. Surgery is the basic method of treatment; complete removal of the meningioma may result in complete recovery [6,7]. Unfortunately, the most common groups are metastatic neoplasms [8], which may constitute up to 40% of tumors located intracranially. The number of patients who experience brain metastases will grow along with improvements in the treatment of primary lesions, leading to the increased survival times of these patients (and an increase in the population over the age of 65) [9,10]. In Poland, so far, little research has been published on the quality of life of people with brain tumors after surgery. Mainly, case reports are published, which is why we prepared a detailed analysis in this aspect.

All above-mentioned intracranially-situated tumors may constitute the causes of numerous disorders. These can include general symptoms that result from augmented intracranial pressures, such as headaches, nausea, vomiting, and altered states of consciousness. Moreover, the locations of these tumors in numerous brain structures result in the appearance of focal symptoms that are characteristic of damage to a given area of the brain [2,11].

Surgery is the basic treatment method for a brain tumor. Unfortunately, only certain brain tumors can be cured by complete resection of the lesion, e.g., grade I gliomas (according to WHO), meningiomas, and neuromas [12]. The aim of surgical treatment (regarding proliferative changes in the brain) is to reduce the tumor mass effect, prevent progression, and maintain neurological functioning [13,14].

The survival of patients with key brain tumors, despite the advanced treatments, is limited; thus, there is ongoing interest in assessing the impacts of treatment on one’s quality of life. The treatments should be carefully selected, taking into consideration the effects on the quality of life of the patients [15].

The quality of life in patients with brain tumors is an issue that is rarely studied in the perioperative period and presented in the available publications. It should be emphasized that patients with brain tumors require hospitalization in specialized clinics equipped with modern technological solutions in this field of medicine, allowing for controlled interventions in sensitive areas of the human body (e.g., the human brain). Patients with brain tumors who qualify for surgical treatments may have clinical symptoms of the disease, but some patients do not have any symptoms of the disease (the only symptom of the disease was, for example, a single seizure). However, the need for intracranial surgery is associated with the risk of neurological symptoms, such as paresis, speech disorders, etc., or their intensifications, which significantly hinder the functioning of these people, and the patients themselves will become dependent on others. Moreover, the histopathological diagnosis of the tumor may significantly affect one’s quality of life. Patients with brain tumors (high-grade glial, metastatic) must undergo adjuvant treatments, which cause great anxiety, and may require further hospitalization, side effects, and economic outlays. Therefore, according to the authors, it is important to undertake quality of life research even before aggressive treatment begins. Hence, we wanted to discover what the quality of life in different groups looked like, e.g., in terms of histopathological diagnosis in the early and late postoperative periods [1,6,11,12,13,14,15].

In summary, the aim of this study was to assess the quality of life after surgical brain tumor procedures.

## 2. Materials and Methods

### 2.1. Study Design and Participants

The research included brain tumor patients scheduled for surgical treatment at the Department of Neurosurgery, the 10th Military Research Hospital at the Health Care Centre Polyclinic in Bydgoszcz. The examinations were performed on the day of admission to the department, on the fifth day after the removal of the brain tumor, and thirty days after the surgery. The exclusion criteria were: no informed consent, lack of sufficient speech, hearing, and cognitive abilities, dementia, or Alzheimer’s disease diagnosed by a psychologist or physician. The research was conducted and analyzed from 2011 to 2019.

**Figure 1:**
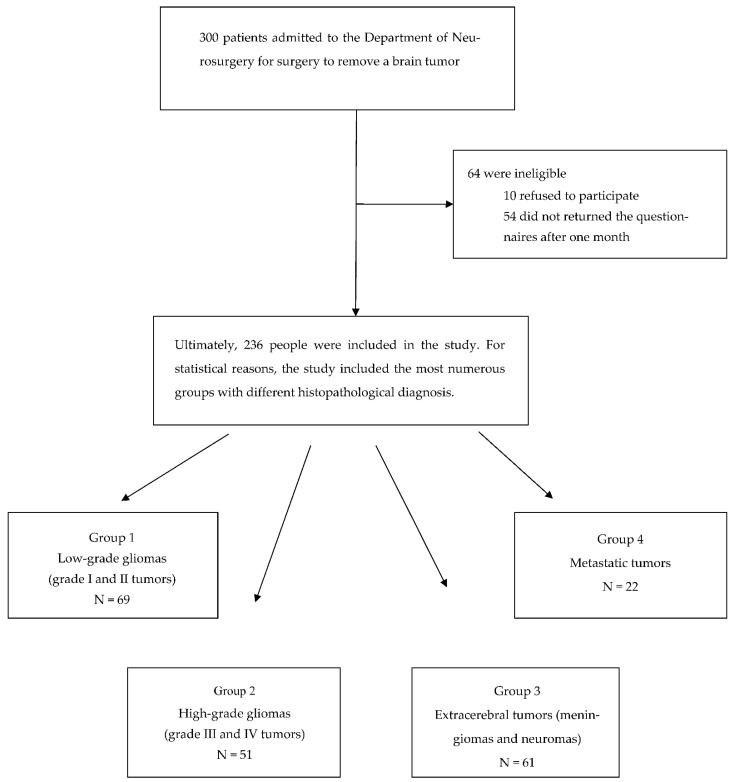
Recruitment flow chart.

Before the procedure, the study group was made up of 300 people—156 women and 144 men aged 16 to 89 (mean age 46.6 ± 15.2 years). After analyzing all stages of the research, 236 patients had full documentation. A total of 64 people were not included in the analysis—10 refused to participate, 54 did not return the questionnaires after one month (Figure 1). Thus, the final study group consisted of 236 people; there was a slight majority of women (52.5%). Table 1 illustrates the baseline characteristics of the participants.

When analyzing the quality of life after the surgical procedures of patients with different histopathological diagnoses, four groups of patients were included—69 patients with low-grade glial tumors (WHO I, II) (group 1), 51 patients with high-grade glial tumors (WHO III, IV) (group 2), 61 patients with tumors that originated from tissues other than the brain, called extracerebral tumors, which included meningiomas and neuromas (group 3), and 22 patients with metastatic tumors (group 4). Groups of tumors with too-small numbers were excluded from the analysis.

### 2.2. Data Collection: Outcome Measures and Procedures

Each patient filled out the following quality of life questionnaires three times (on the day of admission to the neurosurgical department, 5 days after the brain tumor surgery, 30 days after the surgical procedure): the Quality of Life Core Questionnaire of the European Organization for the Research and Treatment of Cancer (EORTC QLQ-C30) version 3.0 and the European Organization for Research and Treatment of Cancer Quality of Life Questionnaire—Brain Module (EORTC QLQ-BN20).

On the day of admission, each patient who qualified for surgery filled out the above questionnaires on their own or with help (in the case of a writing deficit or visual impairment). The second study was carried out on the fifth day of hospitalization; on the day before the patient’s discharge from the neurosurgical clinic, the patient completed the questionnaires on his own or with help, similar to before the surgery. The third examination was performed on the thirtieth postoperative day (before initiation of the adjuvant treatment of the tumor). Before discharge, each patient received questionnaires to be completed within the indicated deadline, along with envelopes, for the questionnaires to be returned to the provided address (the subject was informed the day before the questionnaire was to be completed).

Patient assessment took place 5 days after the brain tumor surgery because patients whose postoperative periods ran smoothly left the Department of Neurosurgery (the clinic where the research was conducted) on the sixth day. Of course, patients who had to be hospitalized for longer periods of time stayed in the department. Performing examinations on patients on the day of discharge would be logistically difficult; it is a very important day for the patients, and sometimes very stressful due to the fact that the patients are provided with important information about further functioning, e.g., with a wound, as well as subsequent consultations with specialists and treatments. Hence, the postoperative fifth day was selected to assess the quality of life. The third examination was carried out on the 30th day after the surgery, because after this date, patients who required adjuvant treatments (radiotherapy, chemotherapy) may have already undergone such treatments that could have significantly affected their quality of life.

EORTC QLQ-C30 consists of 30 questions concerning 5 functional scales (physical, role, cognitive, emotional, and social) and 9 symptom scales (fatigue, nausea and vomiting, pain, dysphonia, insomnia, loss of appetite, constipation, diarrhea, and financial difficulties) [16,17,18]. In turn, EORTC QLQ-BN20 consists of 20 questions and is composed of subscales regarding future uncertainty, visual disorder, motor dysfunction, and communication deficits. In addition, it contains questions regarding headaches, seizures, drowsiness, hair loss, itchy skin, weakness of the legs, and bladder control [19,20]. The assessment ranges of both scales concern the last week of the respondent’s life.

Both questionnaires had requirements for each question (except for questions 29 and 30), ranked on a 4-point Likert scale, which characterized the symptom severity related to the quality of life level. The responses were as follows: 1 = not at all, 2 = a little, 3 = quite a bit, 4 = very much. Higher scores indicated worse health and psychological conditions of the patient. On the other hand, questions 29 and 30, regarding self-assessment of general health and quality of life, ranked the answers on a 7-point scale, from “very bad” (1) to “perfect” (7), i.e., the higher the score, the better the quality of life and health of the patient. The suggested computational method for the quality of life of a given patient was composed of normalizing (bringing all grades to the 0–1 scale) and calculating the final score as the average score of all responses. The quality of life was assessed on a scale from 0 to 1, where 0 indicated a very low quality of life and 1 indicated a good quality of life.

### 2.3. Ethical Statement

The study was approved by the Bioethics Committee of the Nicolaus Copernicus University in Toruń at Collegium Medicum of Ludwik Rydygier in Bydgoszcz, Poland (approval no. 222/2011). The study was conducted as stated by the Declaration of Helsinki in terms of research regarding humans. All subjects provided informed consent in order to participate in the study.

### 2.4. Statistical Analysis

**Figure 2:**
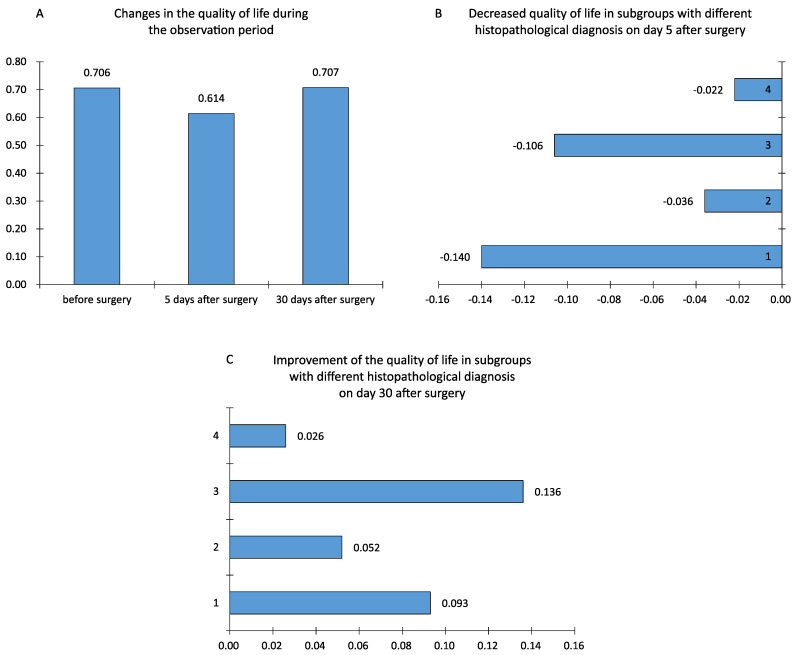
The influence of time and histopathological diagnosis on the quality of life in people with brain tumors.

The collected research material was analyzed in a statistical manner using the Statistica v.12.5 program. The values of the analyzed quantitative parameters are presented as mean (M), standard deviation (SD), and median (Me), while qualitative parameters are presented as numerical or percentage values. Differences in patient characteristics were evaluated with *t*-tests for continuous data (Table 2, Figure 2). The study also used the Shapiro–Wolf non-parametric test aimed at the verification of the hypotheses (concerning the normality of the distributions of the examined features). In the first case, the Shapiro–Wolf test did not lead to rejecting the hypothesis of the normal data distribution from group 4 (i.e., patients with metastatic tumors), while Levene’s test determined the occurrence of differences between the variances (*p* = 0.014). Thus, the Kruskal–Wallis non-parametric test was also used. On the other hand, in the second case, the Shapiro–Wolf test did not lead to rejecting the hypothesis of the normality of the data distribution from group 4; likewise, Levene’s test did not distinguish substantial differences between the variances in the compared groups (*p* = 0.15). This allowed the use of only the one-way ANOVA parametric test (Table 3). Fisher’s least significant difference—LSD post hoc test was used to distinguish significant differences between individual means in more than two groups (Table 4).

## 3. Results

The mean quality of life score evaluated with the EORTC QLQ-30 and EORTC QLQ-BN20 questionnaires from the period before surgery amounted to 0.706, 5 days after surgery it was 0.614, and 30 days after surgery it was 0.707. Moreover, 5 days after the surgical procedure, the quality of life declined, while 30 days after the surgical treatment, it returned to its original state; differences (changes) in the quality of life between individual patient observations were also calculated. The variance between the preoperative period and the fifth day after the surgical procedure was a negative value: −0.091 (*p* < 0.0001). Between the fifth and thirtieth days after the surgery, the quality of life improved—the mean value amounted to 0.093, and it was statistically significant (*p* < 0.0001). Between the first and the third study, the difference turned out to be minimal and statistically insignificant (*p* = 0.91) (Table 2).

On the fifth day after surgery, all groups of patients displayed a reduced quality of life. The utmost decrease in the quality of life after the removal of a brain tumor was observed in patients with low-grade glial tumors (WHO I and II) (M = −0.140) and in the group of patients with extracerebral tumors, such as meningiomas and neuromas (M = −0.106). A smaller reduction in the quality of life was noted in the group of patients with high-grade glial tumors and metastatic tumors. Furthermore, the ANOVA test revealed a crucial difference between the means (*p* = 0.003). The Shapiro–Wolf test did not reject the hypothesis of the normality of the data distribution from group 4, while using Levene’s test led to establishing differences between the variances (*p* = 0.014). For this reason, the Kruskal–Wallis non-parametric test was also applied, which helped confirm the above results (*p* = 0.0025)—the compared groups differed in terms of the deterioration of the quality of life. Each group obtained positive mean lesions thirty days after the surgical procedure, showing that there was a general improvement in the quality of life one month after the brain tumor surgery. The Shapiro–Wolf test did not reject the hypothesis of the normality of the data distribution from group 4; moreover, Levene’s test did not detect significant differences between the variances in the groups under comparison (*p* = 0.15). This allowed the use of the one-way ANOVA parametric test, which found that not all statistical means were equal (*p* = 0.013), meaning that a significant difference between some means was noted (Table 3).

To refine the test results, the post hoc NIR test (least significant difference) was applied. The test illustrated that the decreased quality of life in the group of patients with benign glial tumors and those with extracerebral tumors was significantly greater compared to the other groups. It was also observed that the quality of life improvement between 5 and 30 days after the surgical procedure in the group of patients with benign extracerebral tumors was significantly greater compared to the groups of patients with malignant glial tumors and metastatic lesions. No difference was noted between the mean levels of quality of life in the remaining groups. Thus, patients with extracerebral origin tumors (patients in group 3) showed a significantly greater quality of life improvement between days 5 and 30 after the surgical procedure (Table 4).

Additionally, the most important results from Table 3 and Table 4 are presented in Figure 2.

**Figure 3:**
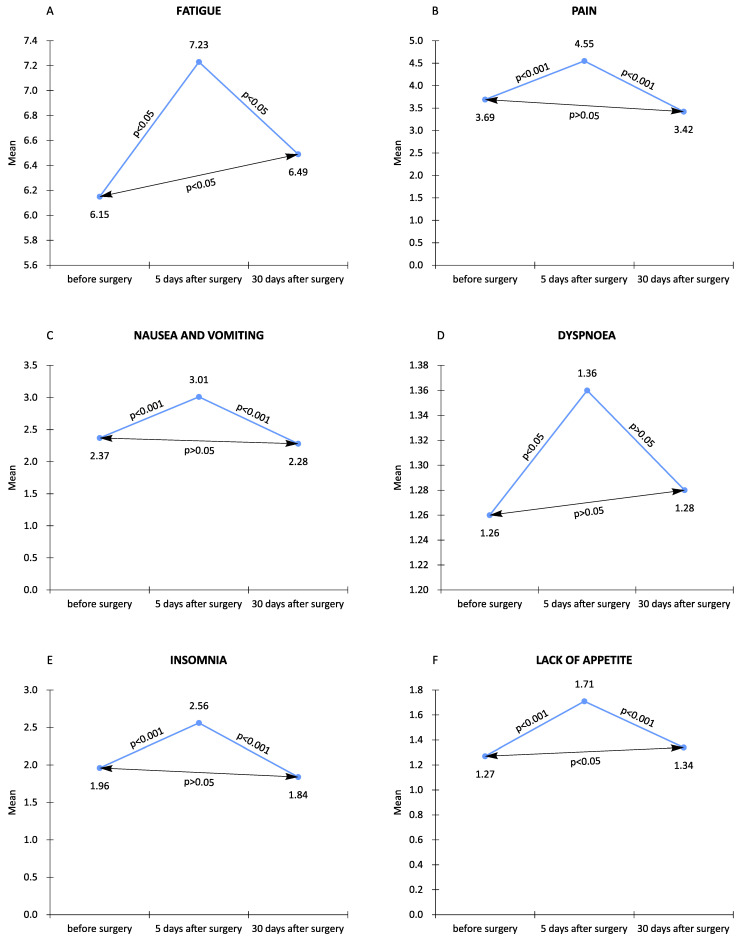
Influence of individual symptoms on quality of life in different time measures.

As a description of the changes in the quality of life, we present the observed changes of several symptoms included in the EORTC QLQ-C30 questionnaire (fatigue, nausea and vomiting, pain, dyspnea, insomnia, lack of appetite). Fatigue was reported at a significant level in the preoperative period; it increased statistically significantly on the fifth day of observation. In turn, on the 30th day, the feeling of fatigue decreased but did not reach the level from before the procedure (*p* < 0.05). Pain occurring in the preoperative period increased in the second observation; in the third observation, it decreased below the preoperative level. The changes in pain were statistically significant (*p* < 0.001). Nausea and vomiting were observed in patients at low levels before the surgery; they intensified in the second period of observation, and the level of perceived symptoms decreased in the third follow-up. These changes were also statistically significant. Dyspnea, a symptom slightly experienced by patients in the preoperative period, intensified statistically significantly (*p* < 0.05) on the fifth day after surgery. Moreover, the level of dyspnea decreased on the 30th day of observation, but it was statistically insignificant (*p* > 0.05). Insomnia was observed at a low level in patients in the postoperative period, worsened in the early postoperative period, and decreased on the 30th day of the follow-up. The changes in the problem were also statistically significant (*p* < 0.001). Lack of appetite was experienced by patients at a low level in the period before the surgery; it increased in the second follow-up period, and decreased on the 30th day of observation—the changes also turned out to be statistically significant (*p* < 0.001). Regarding fatigue and lack of appetite, a statistically significant change was demonstrated between the first and third observations (*p* < 0.05) (Figure 3).

## 4. Discussion

In the approach to the oncological patient, quality of life has become a parameter as important as other parameters characterizing the success of treatment. These days, it is treated on par with figures representing such data as overall survival, disease-free life, and life expectancy with a controlled disease [21].

Malignant brain tumors are generally divided into primary brain tumors (derived from brain tissues) and secondary tumors (metastases). The median survival for primary tumors ranges from several months to several years, while for metastatic tumors, it is several months. Moreover, patients with metastatic neoplasms may suffer from other systemic disorders resulting from the underlying disease. In these cases, the goal of treatment is to alleviate, manage, and prevent complications [22]. Hence, maintaining a good quality of life is a priority in patients with malignant brain tumors.

Surgical treatment of brain tumors is more advantageous compared to other methods, as it allows for establishing a histopathological diagnosis; as a result of a surgical procedure, there is a rapid reduction in the tumor mass, which will diminish or eradicate the patient’s neurological symptoms and cognitive deficits [23]. On the other hand, surgical treatment and perioperative injuries can lead to neurological and cognitive deficits. These deficits can be brief and result in a temporarily reduced quality of life [4].

Nowadays, the assessment of the quality of life is frequently applied in clinical trials as a disease severity indicator or as a result [24,25]. We decided that the perioperative examination is an important parameter that should be checked among our patients who undergo procedures using modern surgical technologies. The instruments used in the authors’ research are well-recognized and commonly used tools for evaluating the quality of life in a multidimensional aspect. The average quality of life assessed using the EORTC QLQ-30 and EORTC QLQ-BN20 questionnaire from the period before the surgery amounted to 0.706, 5 days after surgery to 0.614, and 30 days after surgery to 0.707. Moreover, 5 days after surgery, a significant reduction in the level of the quality of life was observed, while after 30 days, the quality of life significantly improved, reaching the level of the quality of life from before the applied treatment. The quality of life can be reduced by numerous factors. These certainly include all neurological deficits, epilepsy, as well as anxiety and unease related to the procedure and the consequences of the surgical treatment. This was confirmed by the studies by Giovagnoli et al. [25], who revealed significant anxiety among patients in the preoperative examination related to the waiting time for diagnosis. In the studies by Bunevičiuset al. [26], the factors that led to reducing the preoperative quality of life included insomnia, fatigue, headaches, and uncertainty about the future. Cheng et al. [27] analyzed patients with glial tumors of the brain in the preoperative period. When examining the quality of life using the EORTC QLQ-30 questionnaire, the median for the emotional area amounted to 66.7; for the social area to 75.0; for the cognitive area to 83.3; for the physical area to 86.7; and for the functional area to 91.7 (assessing the quality of life on a linear scale from 0 to 100, the higher the value, the better the results). These results indicate that patients had more difficulties in emotional and social areas compared to cognitive, physical, and functional areas.

In the studies by Shin et al. [28], patients with greater functional capacities had significantly better functioning and lower symptom scores in all elements of QLQ-C30 and QLQ-BN20 (lower score—lower severity of a symptom/problem) compared to patients with lower functional capacities on the Karnofski scale. Patients with brain gliomas achieved lower results in terms of physical, cognitive, and social functioning; moreover, there was uncertainty about the future, and motor and communication deficits compared to patients with meningiomas. In the same study, the authors emphasized that patients who underwent brain tumor surgeries alone had a better quality of life compared to patients who underwent surgery and adjuvant treatment (worse functioning, lower quality of life, higher uncertainty about the future, greater communication deficits). The justification for such a result may be the feeling of the severity of the disease by patients treated with combination therapy (which lasts much longer and causes side effects) compared to only-operated patients [28]. In the studies by Jakola et al. [29], no changes in the median EQ-5D indexes were observed following surgery, 0.76 vs. 0.75 (*p* = 0.419). Daily routine activities were significantly altered (*p* = 0.010), which led to a worse outcome after the surgical treatment. No significant changes were noted in dimensions, such as mobility, self-care, pain/discomfort, and anxiety/depression. The authors indicated that the research was performed 6 weeks after the surgery. They emphasized that earlier assessment would be significantly impacted by the short-term postoperative symptoms, while the too-late assessment would be unsuitable due to substantial tumor growth and adjuvant treatment [29]. This could also point to the reduction in the overall quality of life on day 5 after tumor resection, included in our study.

In our own studies, a greater reduction in the quality of life was observed in the group of patients with low-grade glial tumors (WHO I, II) and in the group of patients with extracerebral tumors, such as meningiomas and neuromas; the smallest, on the other hand, was in the group of patients with high-grade tumors (WHO III, IV) and metastatic tumors. Thirty days after surgery, all groups exhibited an increased quality of life. The best quality of life was observed in patients after meningioma and neuroblastoma surgery, and the lowest after metastatic tumor surgery. High-grade primary brain tumors are aggressive tumors; they cause many symptoms, including neurological deficits, but also cognitive impairment, which may remain at a similar level after surgery. The situation is similar in patients with metastases to the brain and disorders caused by the underlying disease (the brain tumor may remain at a similar level). In the case of lower-grade primary neoplasms, deficits may appear only after surgery, but, importantly, as a result of the implemented anti-edematous treatment and rehabilitation, they disappear, so it is a temporary state. Hence, in these groups of patients, the quality of life significantly decreases in the early postoperative period. In the studies by Salo et al. [30], patients with malignant gliomas (WHO grade III and IV) exhibited the lowest quality of life in the preoperative period. Chiu et al. [22] reviewed the literature on the quality of patients with primary and metastatic brain tumors. After the analysis, the authors concluded that patients with primary brain tumors are characterized by better social and functional well-being compared to patients with secondary tumors. The remaining elements of the quality of life were comparable. In another report, Chiu et al. [31] also performed a comparison of differences in the quality of life in patients with primary brain tumors and metastatic tumors assessed with the QLQ-BN20 and QLQ-C30 questionnaires. The performance statuses of the patients in both groups were at analogous levels. Patients with primary brain tumors and brain metastases were characterized by the following values: physical functioning—weighted average: 79.18 and 74.93, overall quality of life—61.88 and 59.44, role functioning—67.37 and 75.00, and emotional functioning—70.44 and 71.86. The values were not statistically significant. Only cognitive functions (zQLQ-C30) were assessed at significantly worse levels in patients with primary brain tumors (*p* = 0.0199). Despite this, quality of life profiles were very similar among the patients with metastatic tumors and primary tumors. In the study by Shin et al. [28], patients with gliomas (39.7%) had significantly lower physical, cognitive, and social functioning along with higher uncertainty of the future, movement disorders, and communication disorders compared to patients with brain meningiomas *(p* *<* 0.001; −0.02). Tsay et al. [32], in studies conducted on a group of benign brain tumor patients, did not demonstrate any significant changes in the quality of life 1 month after the surgical treatment. On the other hand, Jakola et al. [29] concluded that modern neurosurgical treatments do not have any impact on the quality of life in patients with brain tumors, and the improvement in the quality of life is not a result of this procedure. They emphasize that new deficits should be avoided at any cost, as they may have serious, undesirable impacts on the quality of life in patients with brain tumors. Our research confirms the above opinions.

This study has some limitations. First, the study was conducted in only one hospital in the city. Consequently, it may not be representative of all hospitalized brain tumor patients nationwide. Secondly, the study did not take into account factors such as the occurrence of complications after surgery or the length of stay in the ward, which could significantly affect the assessed quality of life. Thirdly, 30 days post-operatively is a short time period. It should be considered that 30 days is not long-term. Therefore, it is necessary to continue longitudinal multicenter studies throughout the country.

The study did not take into account complications after surgery, because the tools themselves took into account questions about symptoms (problems) that may have also been consequences of surgery. In many cases, they were temporary and affected the quality of life only in the early postoperative period (5 days after surgery). When assessing patients on the 30th day after surgery, their quality of life improved (returning to the level from before the surgery), because the deficits that occurred early after the surgery had subsided and the stress associated with surgery or waiting for a histopathological result had disappeared. 

It was assumed that the patients were examined after the surgery on the 5th and 30th days. The hospitalization times of patients at the clinic were not taken into account in the study. The study excluded patients with whom it was not possible to conduct the study (i) due to their health conditions, (ii) to complete the required tools within the specified days, or (iii) because patients did not return the completed questionnaires.

## 5. Conclusions

To summarize, the lowest quality of life was recorded on day 5 after surgery. Many symptoms, such as fatigue, nausea and vomiting, pain, dyspnea, insomnia, and lack of appetite, intensified (especially on the 5th day after surgery). Furthermore, the histopathological diagnosis of the tumor impacted the quality of life. In the first postoperative period, a decreased quality of life was observed in the group of low-grade glial neoplasms and benign tumors, such as meningiomas and neuromas. In contrast, 30 days after the surgical treatment, the lowest quality of life was recorded in patients with metastatic tumors.

## Figures and Tables

**Table 1:** The sociodemographic characteristics of the participants.

		*n*	%
Gender	Woman	124	52.5
	Man	112	47.5
Age	<21 years	9	3.8
	21–40 years	75	31.8
	4–60 years	103	43.6
	>60 years	49	20.8
Place of residence	Village	72	30.5
	City up to 25,000 residents	49	20.8
	City from 26,000 to 100,000 residents	36	15.3
	City over 100,000 residents	79	33.5
Education	Primary	16	6.8
	Vocational	59	25.0
	Secondary	89	37.7
	Higher	72	30.5
Marital status	Single	38	16.1
	Married/in a partnership	176	74.6
	Divorcee	11	4.7
	Widower/widow	11	4.7
Professional status	Student	14	5.9
	Working professionally	105	44.5
	Pension	91	38.6
	Pension plus working professionally	14	5.9
	Unemployed	12	5.1

**Table 2:** Quality of life after brain tumor surgery.

	Observation Periods	Min	Max	Median	Mean	SD	Statistical Result
Quality of life	Before surgery	0.16	1.00	0.71	0.706	0.150	Df2 = 67.74920 *p* = 0.000 *
	5 days after surgery	0.13	0.99	0.63	0.614	0.169	
	30 days after surgery	0.17	1.0	0.72	0.707	0.158	
Changes in the quality of life during the observation period	Preoperative period—5th day after surgery	−0.70	0.41	−0.10	−0.091	0.181	t = 7.72 *p* < 0.0001 **
	5 days after surgery—30 days after surgery	−0.41	0.57	0.10	0.093	0.168	t = 8.50 *p* < 0.0001 **
	Preoperative period—30 days after surgery	−0.45	0.38	−0.01	0.001	0.140	t = 0.11 *p* = 0.91 **

*—Friedman test; **—Student’s *t*-test; SD—standard deviation.

**Table 3:** Changes in the quality of life after surgery in groups with different histopathological diagnoses.

Histopathological Diagnosis of a Brain Tumor	*n*	Min	Max	Median	Mean	SD	Statistical Result
Changes in the quality of life between the examination before the operation and the 5th day after the operation							
Low-grade gliomas (grade I and II tumors)	69	–0.75	0.31	–0.15	–0.140	0.153	* F = 4.70 *p* = 0.003 ** H = 14.3 *p* = 0.0025
High-grade gliomas (grade III or IV tumors)	51	–0.45	0.41	–0.05	–0.036	0.166	
Extracerebral tumors—meningiomas, neuromas	61	–0.61	0.33	–0.09	–0.106	0.212	
Metastatic tumors	22	–0.28	0.30	–0.03	–0.022	0.151	
Changes in the quality of life between the 5th postoperative day and the 30th postoperative day							
Low-grade gliomas (grade I and II tumors)	69	–0.412	0.572	0.073	0.093	0.162	* F = 3.66 *p* = 0.013
High-grade gliomas (grade III or IV tumors)	51	–0.289	0.300	0.074	0.052	0.142	
Extracerebral tumors—meningiomas, neuromas	61	–0.313	0.456	0.126	0.136	0.169	
Metastatic tumors	22	–0.318	0.325	0.064	0.026	0.194	

* Analysis of variance (ANOVA); ** Kruskal–Wallis test.

**Table 4:** Changes in the quality of life after surgery in groups with different histopathological diagnoses, refining the test results using a post hoc NIR test (checking which means differ significantly from each other).

Five days after surgery	Histopathological diagnosis of a brain tumor	1	2	3	4
		Mean = −0.1398	Mean = −0.0363	Mean = −0.1060	Mean = −0.0223
		*p*	*p*	*p*	*p*
	1—Low-grade gliomas (grade I and II tumors)		0.002 *	0.275	0.007 *
	2—High-grade gliomas (grade III or IV tumors)	0.002 *		0.038 *	0.775
	3—Extracerebral tumors—meningiomas, neuromas	0.275	0.038 *		0.056
	4—Metastatic tumors	0.007 *	0.755	0.056	
Thirty days after surgery		Mean = 0.09346	Mean = 0.05213	Mean = 0.13613	Mean = 0.02605
		*p*	*p*	*p*	*p*
	1—Low-grade gliomas (grade I and II tumors)		0.172	0.139	0.094
	2—High-grade gliomas (grade III or IV tumors)	0.172		0.007 *	0.532
	3—Extracerebral tumors—meningiomas, neuromas	0.139	0.007 *		0.007 *
	4—Metastatic tumors	0.094	0.532	0.007 *	

*—significant dependencies; *p*-value—the post hoc Fisher’s least significant difference test.

## Data Availability

The data presented in this study are available upon request from the corresponding author. The data are not publicly available due to respondents privacy.
